# The Public Health 12 framework: interpreting the ‘Meadows 12 places to act in a system’ for use in public health

**DOI:** 10.1186/s13690-022-00835-0

**Published:** 2022-03-07

**Authors:** Kristy A. Bolton, Jillian Whelan, Penny Fraser, Colin Bell, Steven Allender, Andrew D. Brown

**Affiliations:** 1 Institute for Physical Activity and Nutrition, School of Exercise and Nutrition Sciences, Deakin University, Geelong, Australia; 2 Institute of Health Transformation, School of Health and Social Development, Global Obesity Centre, Deakin University, Geelong, Australia; 3 Institute of Health Transformation, School of Medicine, Global Obesity Centre, Deakin University, Geelong, Australia

**Keywords:** Systems science, Systems thinking, Public health, Leverage points, Implementation

## Abstract

**Background:**

Systems science approaches have demonstrated effectiveness in identifying underlying drivers of complex problems and facilitating the emergence of potential interventions that are locally tailored, feasible, sustainable and evidence informed. Despite the potential usefulness of system dynamics simulation modelling and other systems science modelling techniques in guiding implementation, time and cost constraints have limited its ability to provide strong guidance on how to implement complex interventions in communities. Guidance is required to ensure systems interventions lead to impactful systems solutions, implemented utilising strategies from the intersecting fields of systems science and implementation science. To provide cost-effective guidance on how and where to implement in systems, we offer a translation of the ‘Meadows 12 places to act in a system’ (Meadows 12) into language useful for public health.

**Methods:**

This translation of Meadows 12 was informed by our experience in working with 31 communities across two complex large scale randomised control trials and one large whole of community case study. These research projects utilised systems science and implementation science to co-create childhood obesity prevention interventions. The team undertaking this translation comprised research academics, implementation specialists and practitioners, practice-based researchers and a systems dynamicist. Our translation of each of the Meadows 12 levels to act in the system maintains the fidelity and nuance of the 12 distinct levels. We provide examples of each level of the Public Health 12 framework (PH12) drawn from 31 communities. All research was conducted in Victoria, Australia between 2016 and 2020.

**Results:**

PH12 provides a framework to guide both research and practice in real world contexts to implement targeted system level interventions. PH12 can be used with existing implementation science theory to identify relevant strategies for implementation of these interventions to impact the system at each of the leverage points.

**Conclusion:**

To date little guidance for public health practitioners and researchers exists regarding how to implement systems change in community-led public health interventions. PH12 enables operationalisation Meadows 12 systems theory into public health interventions. PH12 can help research and practice determine where leverage can be applied in the system to optimise public health systems level interventions and identify gaps in existing efforts.

**Trial registration:**

WHO STOPS: ANZCTR: 12616000980437. RESPOND: ANZCTR: 12618001986268p.

## Contributions to the literature

- Several studies are now published that use systems methodologies to understand complex problems. Very few of these studies identify *how* and *where* to act in a system and those that do apply reduced versions of Meadows’s original 12 places to intervene in a system, thereby losing opportunity for maximum system impact.
- The Public Health 12 Framework presented here provides specific guidance on how to use Meadows’s ground-breaking framework in public health interventions whilst maintaining fidelity to the original systems science constructs.

## Background

Systems science has been applied to varied complex problems including physics and climate change [1], and more recently to address complex problems in public health such as obesity [2]. Advantages include providing a method to simultaneously consider and navigate the social ecological model of health [3]. Part of the power of using systems science to tackle complex health problems is that subsequent interventions are designed to be adaptive and provide space for multiple solutions to emerge [4].

Community-based system dynamics (CBSD) [5], and embedded techniques like group model building (GMB), are participatory, capture mental models of problems and model impacts of intervention effectiveness [5–8]. CBSD aligns with and builds on community-based prevention efforts which emphasise community capacity building [9–11], engagement and knowledge sharing [5]. In GMB, groups work with a facilitator to build a shared mental model of the causes of a problem from a local perspective [12]. From this shared model, participants can then identify places to act, relevant interventions and related implementation strategies adapted to the local context [13, 14]. Evidence of the effectiveness of GMB to build a common understanding of complexity and identify interventions is growing [8, 15].

Our understanding of which interventions or combination of actions are most influential to impact systems change is less advanced. Donella Meadows was a pioneer in system dynamics and engineering and proposed 12 places to intervene in a system for maximum impact [16], referred to as Meadows 12 (M12) (see Table 1). The original development of the M12 is described elsewhere, [16] but in brief, they were brainstormed in response to perceived flaws in the World Trade Organisation and other trade deals; and Meadows’ own experiences with systems thinking in general and system dynamics modelling in particular. The ideas were further refined prior to publication, but in the conclusion of the article, they are described as tentative, creating an invitation for further development [16]. System dynamics simulation modelling has been used as an implementation science tool in healthcare and health promotion [17], demonstrating its usefulness in adopting a systems approach to implementation science. However, given the cost and time required for simulation, there is a need to fast-track insights generated from simulation into community-based implementation. M12 is frequently referenced as a tool to translate these insights from system dynamics modelling. To date these insights are underutilised because of the difficulty in translating systems language to public health language, as demonstrated by multiple attempts to simplify the framework for use in public health [18, 19].

**Table 1:** Comparison between the original definition of Meadows 12 [16] with the translation specific to public health in Public Health 12

Level	Meadows 12 (M12) [16]	Public Health 12 (PH12)
1. Transcending Paradigms	The power to transcend paradigms	The ability to continually adapt collective fundamental beliefs leading to widespread change in the way things are, to respond effectively to multiple complex problems.
2. Paradigms	The mind-set out of which the system – its goals, structure, rules, delays, parameters – arises	A population-level shift in fundamental beliefs (e.g. cultural shift) on how to respond effectively to complex problems (a change in the way things are).
3. Goals	The purpose or function of the system	Where a fundamental goal of a system is challenged and changed.
4. Self-organisation	The power to add, change, or evolve system structure	Creating and maintaining infrastructure (e.g. political or governance) for implementing a combination of various level 5-12 actions over time.
5. Rules	Incentives, punishments, constraints	New modified rules such as incentives and accountability mechanisms for change.
6. Information flows	The structure of who does and does not have access to information	Movement of vital information to shift power dynamics that opens the decision-making processes to more (and the right) people.
7. Reinforcing feedback loops	The strength of the gain of driving loops	Initiating a movement toward a target that is self-reinforcing and growing exponentially in the desired direction.
8. Balancing feedback loops	The strengths of the feedbacks relative to the impacts they are trying to correct	Taking action to stabilise a part of the system to achieve a specific intended goal.
9. Delays	The lengths of time relative to the rates of system changes	Strategic planning to align timeframes with available resources, current readiness, and intended outcomes.
10. Stock and flow structures	Physical systems and their nodes of intersection	Building of new physical infrastructure, providing financial infrastructure, and/or improving physical movement through the system.
11. Buffers	The sizes of stabilizing stocks relatives to their flows	To maintain a safety net within our community or system to absorb reasonably foreseeable, but unexpected events without adversely affecting the way things are. This includes supports for individuals and groups built into environments, schools, workplaces.
12. Numbers	Constants and parameters such as subsidies, taxes, standards	To increase or decrease one isolated, existing part of the system.

The leverage points are presented in order of their potential to create an impact from small changes to existing structures to the power to transcend paradigms. The M12 has been critiqued for its technical language and subsequent difficulty in translating to fields outside engineering [19, 20]. While others have adapted the M12 [18–20], their approach has been to collapse it into fewer levels, losing some of the nuance and fidelity to the original framework. We set out to translate M12 into language that maintains the structure of the original M12 and is specifically tailored for use by public health practitioners and researchers working on public health interventions, such as obesity prevention.

### Aims

1. To translate the M12 system intervention points into language useful for public health whilst maintaining fidelity to the 12 levels.
2. To provide examples of the intervention points from three large-scale participatory community-based obesity prevention interventions conducted in Victoria, Australia 2016-2020.

## Methods

### Context

Building on previously reported community-based approaches to obesity prevention [9, 11], the Global Obesity Centre (GLOBE) at Deakin University has been trialling CBSD to empower communities for childhood obesity prevention since 2014 [21]. Two recent trials include the Whole of Systems Trial of Prevention Strategies for Childhood Obesity (WHO STOPS) [22] and Reflexive Evidence and Systems interventions to Prevent Obesity and Non-communicable Disease (RESPOND) [23]. These are stepped wedge randomised control trials implemented over 4 years in regional Victoria, Australia; with five intervention communities implementing the intervention in year 1, and the other five communities in year 2. Communities in WHO STOPS (potential population reach 125,000) and RESPOND (potential population reach 213,600) were geographically bounded by local government areas, with the option of refinement of boundaries (i.e., by splitting a geographical area into two or more communities) based on community feedback. The outcomes of WHO STOPS are reported elsewhere [22] and the RESPOND trial is underway [23]. The third study, Yarriambiack – Creating Healthy, Active, Nourished Generations (YCHANGe), potential population reach 7026) was a whole of community obesity prevention initiative implemented in rural Victoria, Australia. The initiative was community-led with researcher oversight for 5 years, with some initiatives sustained long-term [24].

Each study used GMB informed by the scientific evidence base [12, 15] to build a causal loop diagram (CLD) that modelled a shared understanding of the issue. From this, multiple places to intervene in the system were identified along with relevant strategies, consensus and commitment to action [6, 15]. This co-creation of interventions and strategies represented a step beyond standard practice of implementing and testing a pre-defined program of activities and deliberately built community capacity in systems approaches to prevention.

### Translating Meadows 12 definitions into public health language

Six members of the core research team (consisting of academic professors, implementation specialists, practice-based researchers, implementation practitioners and a systems dynamicist) initially reviewed the literature and compared the application and evaluation of existing public health system dynamics frameworks [25, 26]. We purposefully included expertise from both system dynamics and public health to maintain fidelity to both disciplines. PF previously held a community development role for 6 years and has spent the last 5 years as an implementation specialist building community capacity; ADB is a system dynamacist with 8 years in community-based prevention work and simulation modelling; JW has worked for > 20 years in health promotion prior to spending the last 10 years working in academia on community-based obesity prevention; KAB has led the evaluation of large complex state-wide community-based obesity prevention interventions > 10 years and has spent the last 4 years as an implementation specialist in community-based systems approaches to childhood obesity. CB has 27 years of research experience in community-based obesity prevention and public health medicine. SA has over 20 years of research experience in community-based prevention, and 10 years specifically working with communities utilising systems methodologies, and co-developed innovative software for use in building CLDs and reporting actions over time.

Development of the definitions was iterative. Initially the core team (ADB, KAB, JW, PF) provided a draft translation. This was presented to CB and SA for feedback and re-worked. The core team then mapped actions to the draft translations and found several items lacked clarity. Wording was again altered and further input from CB and SA was sought. In our third iteration, we invited input from an external reviewer with experience that spans mental health and wellbeing, physical activity, and disability. This external reviewer had not been involved in the translation process. This process was designed to test alignment between an external party actions mapped by the external reviewer with those of the core research team. This identified key ‘gaps’ in framework alignment which led to a fourth iteration and re-wording, followed by a further iterative round of expert consultation. What is presented here is the fifth iteration of our translation. The translations are focussed on public health because all authors currently work in public health, and public health projects were used to trial the translation.

Twenty-five meetings (each ~ 2 h duration) were conducted between 21/09/2020 and 31/05/2021 with the core research team. First, definitions of M12 were carefully considered. Drawing from both practitioner expertise with participatory community-based prevention work and academic expertise, new consensus translations (definitions) were constructed based upon each level of M12. We then identified examples of each intervention point.

### Testing the new language/proof of concept

To demonstrate the types of actions that sit under the Public Health framework (PH12) leverage points, action registers from three independent community-based systems approaches addressing obesity conducted in Victoria were examined by the author team described above.

The action registers were spreadsheets of documented actions implemented in each community as recorded by the project officer. In keeping with the philosophy of co-designed actions, each community was encouraged to register actions that were important to them. An implementation specialist embedded in the community (PF) coded actions against the PH12. A diverse range of actions were purposefully selected from a pool of over 300. These were presented back to the core research team in a matrix to discuss. This matrix consisted of M12, community, action, concepts for new public health language and comments were noted from discussion between the research team. Continual referral to the original M12 framework and example actions as described was conducted. A consensus agreement was made within the team on which of the 12 leverage points each action best aligned, and which public health language best described the leverage point.

## Results

Table 1 captures our public health translation of the Donella Meadows 12 system leverage points [16].

Example actions of the leverage points are shown in Table 2. Future work will map ~ 200 full actions to the PH12.

**Table 2:** Translation of PH12 with examples from real life implementation of interventions to improve public health

Level	PH12	Example (WHO STOPS [22], RESPOND [23], YCHANGe [24])
1	The ability to continually adapt collective fundamental beliefs leading to widespread change in the way things are, to solve multiple complex problems.	No real-world examples of Level 1 were identified within our interventions, so therefore we provide an aspirational example. A local community experiences multiple environmental crises of bushfires and floods, followed by COVID-19 pandemic. In response to recognising the multiple interconnected complex crises, the community devises a co-created intervention to protect local forests and farmland in a co-ordinated way and simultaneously enhances large scale emphasis of locally sourced food into food systems. This type of triple duty action [4] would impact environment sustainability, community resilience, and public health.
2	A population-levelshift in fundamental beliefs on how complex problems are solved (a change in the way things are).	WHO STOPS: A local water authority champion addressing the taste of tap water at a regional level using a systems approach. – ‘Great Tasting Water Project’. An organisation expanded their remit from providing safe drinking water to providing safe drinking water that people want to drink.
3	Where a fundamental goal of a system is challenged and changed.	YCHANGe and WHO STOPS: When the traditional goal of a health service shifts from treating illness, to keeping people well and in their homes and community and this is written into the mission statement of the health service(s) and enacted in daily practice through multiple actions.
4	Governance structures to create and maintain consensus on a combination of various level 5-12 actions over time.	RESPOND: Established a ‘healthy schools’ collective rather than working with each school separately.
5	New modified rules that incorporate and bring accountability for change.	WHO STOPS: The grouping of water related policies at junior sport; sport stadium not selling sugar sweetened beverages drinks; junior basketball endorsing a “water only policy”.
6	Movement of vital information to shift power dynamics that opens up the decision making processes to more (and the right) people.	WHO STOPS and RESPOND: Sharing of the local child anthropometric data was an initial catalyst for change.
7	Initiating a movement toward a target that is self-reinforcing and gaining momentum in the desired direction.	WHO STOPS, RESPOND and YCHANGe: STOPS: Those in authority positions provided the authority and resources to work in a way that prioritises childhood obesity prevention ongoing.
8	Taking new action to stabilise a part of the system to achieve a specific intended goal.	WHO STOPS: A rural health service recognised the food it provided in its in-house cafe was not healthy. To try to limit or slow the purchase of unhealthy foods, a ‘green only’ (i.e. healthy) food policy was implemented.
9	Strategic planning to align timeframes with available resources, current readiness, and intended outcomes.	YCHANGe: The CEO of the health service strategically providing funding aligned with a new café development and policy mandate to meet an established ‘healthy food’ quota.
10	Building of new physical or providing financial infrastructure and/or improving physical movement through the system.	YCHANGe: new drinking fountains were installed in towns where no public access to free fresh water previously existed.
11	To maintain a ‘safeguard/safety net’ within our community or system to absorb reasonably foreseeable, but unexpected events without adversely affecting the way things are. This includes maintaining supportive environments, schools, workplaces	RESPOND: several organisations pooled financial resources to enable a project officer to be employed in one community to solely work on RESPOND.
12	To increase or decrease one isolated part (component) of the system that already exists within a community?	YCHANGe: Produced a brochure of the current physical activities available and the LGA put a sugar content of soft drinks sign on the fridge.

*CEO* Chief executive officer, *LGA* Local government area, *RESPOND* Reflexive Evidence and Systems interventions to Prevent Obesity and Non-communicable Disease [23], *WHO STOPS* Whole of Systems Trial of Prevention Strategies for Childhood Obesity [22], *YCHANGe* Yarriambiack – Creating Healthy, Active, Nourished Generations [24]

## Discussion

We translated the M12 framework for public health to enable public health practitioners and researchers to categorise actions according to level of impact.

Multiple attempts across various disciplines have adapted the M12 to contemporary study areas [27] though typically with academic, discipline specific language. The Intervention Level Framework (ILF) acknowledged the difficulty of applying M12 to public health and collapsed them into the five level ILF framework [18, 20]. The ILF comprises (from highest to lowest impact): paradigm, goals, system structures, feedbacks and delays, and structural elements. Specific to school settings, McIssac [28] further summarised data aligned to the ILF into three themes of intervention points within the school food system (from highest to lowest impact): purpose and values, system regulation and interconnections, and actors and elements. The ILF has been used successfully utilised to code pre-conference reading, and high-level documents (i.e. government, health, medical reports) related to obesity efforts/strategies to help influence future policy and planning [18]. However, we found no publications where researchers utilised the ILF to categorise system level obesity prevention actions implemented in real world interventions although some work is pending [29]. Nobles et al. [19] challenged the reductionist approach inherent in the ILF and proposed the Action Scales Model (ASM), which aligns with both the ILF and M12. The four levels of the ASM are: beliefs (levels 1 and 2 in M12), goals (level 3 in M12), structures (levels 4-9 in M12), and events (levels 10-12 in M12). The strength of the ASM compared to the ILF is it considers comprehensively how its levels interact and combine to create public health change. However, the ASM shares the same drawback in the ILF in that by reducing M12 to four categories for the sake of simplification, nuanced insights from the original M12 are lost.

Given the work of Malhi [20], Johnston [18], McIssac [28] and Nobles [19] to operationalise M12 with the specified intent to improve usability for public health and policy practitioners with each iteration, why create something new? We argue that we have not created something new but translated the M12 into language useful for public health practice. Rather than reduce the number of levels, we embraced the challenge and the complexity of M12, and the difficulty of interpreting and operationalising the work of a system dynamics expert from a non-public health field into obesity prevention. To deliberately maintain the finesse and nuance of the 12 levels of M12, we engaged with cross-sector expertise. We consider much of the nuance of the original M12 is lost in other translations. This loss of nuance may assist in mapping actions in the short term but may hinder capacity to measure change over time, to fully understand the different impacts of various level actions, and to perform a deep evaluation of what drives systems change.

Another potential advantage of PH12 is helping communities decide ‘what’ to implement once actions have been identified. By offering more levels, communities and practitioners can align and prioritise actions more comprehensively than with the other available systems scales (i.e., choosing between level 4, 5, 6, 7, 8, or 9 actions rather than picking between a big group of actions at the “Structures” level in the ASM). In this way PH12 intersects with the growing field of implementation science, defined as “the scientific study of methods to promote the systematic uptake of research findings and other [evidence based practices] EBPs into routine practice …” [30].

### Public health implications

Durham [31] utilised the ILF to explore systems change in Aboriginal and Torres Strait Islander ear health. We concur with Durham et al. [31] that the implementation of system science would be advanced through an identification of the number of actions and the levels of actions required to facilitate systems change. We consider by maintaining 12 levels, we provide a more nuanced opportunity for such analysis. The PH12 definitions will support public health practitioners and researchers to examine the gaps in implementation by identifying ‘levels’ where no action is planned.

### Strengths and limitations

Strengths of this study include the diverse team experience (i.e., practitioner in community, systems dynamicist, researcher (implementation and evaluation) lenses) with community-based systems approaches to obesity prevention. The PH12 translations were informed by expertise drawn from related fields such as social work, community development and allied health, potentially widening the reach of the proposed PH12. Having an external public health reviewer test the translation provided confirmation that PH12 could be used without being an implementation expert. Testing the framework outside of public health is beyond the scope of the current paper; and testing outside research-based settings is planned. We, like Meadows, offer this as a step forward in the identification of targeted actions for systems change and invite ongoing critique and improvements with other researchers and practitioners.

This newly translated PH12 framework is user friendly and will be a guiding tool that practitioners, researchers, key community stakeholders and policy makers can use to decide where to invest time, effort, and resources. Future work rigorously testing the framework by other CBSD theorists and practitioners, public health researchers and practitioners on the ground implementing community-based systems thinking approaches to address complex problems is recommended and we encourage external validation by experts outside of Australia and in other fields.

## Conclusions

To date little guidance for practitioners and researchers exists regarding where to target actions for community-led public health action such as obesity prevention. We have translated M12 into PH12 to allow improved operationalisation of the 12-level framework through a public health lens. Potentially, a deeper understanding of the potential consequences of action to address complex health problems can be achieved.

## Data Availability

The datasets used and/or analysed during the current study are available from the corresponding author on reasonable request.

## Abbreviations

ASM: Action Scales Model
CBSD: Community-based system dynamics
CLD: Causal loop diagram
GLOBE: Global Obesity Centre
GMB: Group model building
ILF: Intervention Level Framework
M12: Meadows 12
PH12: Public Health 12 framework
RESPOND: Reflexive Evidence and Systems interventions to Prevent Obesity and Non-communicable Disease
WHO STOPS: Whole of Systems Trial of Prevention Strategies for Childhood Obesity
YCHANGe: Yarriambiack – Creating Healthy, Active, Nourished Generations
